# Identification of a Group of GABAergic Neurons in the Dorsomedial Area of the Locus Coeruleus

**DOI:** 10.1371/journal.pone.0146470

**Published:** 2016-01-19

**Authors:** Xin Jin, Shanshan Li, Brian Bondy, Weiwei Zhong, Max F. Oginsky, Yang Wu, Christopher M. Johnson, Shuang Zhang, Ningren Cui, Chun Jiang

**Affiliations:** Department of Biology, Georgia State University, Atlanta, Georgia, United States of America; Baylor College of Medicine, UNITED STATES

## Abstract

The locus coeruleus (LC)-norepinephrine (NE) system in the brainstem plays a critical role in a variety of behaviors is an important target of pharmacological intervention to several neurological disorders. Although GABA is the major inhibitory neurotransmitter of LC neurons, the modulation of LC neuronal firing activity by local GABAergic interneurons remains poorly understood with respect to their precise location, intrinsic membrane properties and synaptic modulation. Here, we took an optogenetic approach to address these questions. Channelrhodopsin (ChR2) in a tandem with the yellow fluorescent protein (YFP) was expressed in GABAergic neurons under the control of glutamic acid decarboxylase 2 (GAD2) promoter. Immediately dorsomedial to the LC nucleus, a group of GABAergic neurons was observed. They had small soma and were densely packed in a small area, which we named the dorsomedial LC or dmLC nucleus. These GABAergic neurons showed fast firing activity, strong inward rectification and spike frequency adaptation. Lateral inhibition among these GABAergic neurons was observed. Optostimulation of the dmLC area drastically inhibited LC neuronal firing frequency, expanded the spike intervals, and reset their pacemaking activity. Analysis of the light evoked inhibitory postsynaptic currents (IPSCs) indicated that they were monosynaptic. Such light evoked IPSCs were not seen in slices where this group of GABAergic neurons was absent. Thus, an isolated group of GABAergic neurons is demonstrated in the LC area, whose location, somatic morphology and intrinsic membrane properties are clearly distinguishable from adjacent LC neurons. They interact with each and may inhibit LC neurons as well as a part of local neuronal circuitry in the LC.

## Introduction

The locus coeruleus (LC) is an isolated nucleus in the pons, deriving >90% norepinephrinergic (NE) neurons in the central nervous system (CNS) [1]. Changes in spontaneous firing activity of the LC neurons by both intrinsic membrane properties and synaptic inputs are known to affect various behaviors and physiological functions, including attention, anxiety, breathing, arousal state, motor function, etc [2–6].

LC neuronal activity is inhibited by γ-aminobutyric acid (GABA) afferents. The alteration of GABAergic inputs under physiological or pathophysiological conditions can lead to distinct LC neuronal firing patterns as reported in a number of previous studies. For example, an increased GABA release is responsible for lower firing frequency of LC neurons in REM sleep, which can be abolished by GABA_A_ receptor antagonists [5, 6]. Certain diseases such as Rett syndrome can cause dramatic defects in both pre- and postsynaptic GABAergic systems contributing to the hyper-excitability of LC neurons [7, 8].

Although previous studies have shown that several brain regions provide GABAergic inputs to the LC including medullary nuclei and forebrain [9, 10], local neuronal networks in the LC area remain elusive, especially GABAergic inhibition. It is still unclear whether in the LC region GABAergic neurons form isolated groups, what intrinsic properties the GABAergic neurons have, and how they interact with each other. The major obstacle to study these local GABAergic neurons is the lack of cell-specific identifications that allow unambiguous electrophysiological recordings.

The optogenetic approach provides a unique opportunity to overcome this obstacle. Therefore, we took the advantage of commercially available GAD2-Cre and LoxP-channelrhodopsin (ChR2) mice, and expressed ChR2 with the yellow fluorescent protein (YFP) in GABAergic neurons [11]. Using these mice, we identified a group of GABAergic neurons in the vicinity of dorsomedial LC, revealed their intrinsic properties, found their synaptic inhibition to each other, and saw some evidence for the inhibition of LC neurons by these GABAergic neurons.

## Materials and Methods

### Transgenic animals

All experimental procedures in the animal were conducted in accordance with the National Institutes of Health (NIH) *Guide for the Care and Use of Laboratory Animals* and were approved by the Georgia State University Institutional Animal Care and Use Committee. Transgenic mice were generated by cross-breeding the strain of GAD2-Cre mice (*Gad2*^*tm2(cre)Zjh*^*/J*, Jackson Laboratory SN 010802) with the ChR2-eYFP-LoxP strain (*B6;129S-Gt(ROSA)26Sor*^tm32(CAG-COP4*H134R/EYFP)Hze^*/J*, Jackson Laboratory SN 12569). The offspring were routinely genotyped with a PCR protocol provided by the Jackson Laboratory. Only male animals were used in the present study.

### Preparation of brain slice

Brain slices were prepared as described previously [7, 8]. In brief, animals at 3 weeks of age were anesthetized with inhalation of saturated isoflurane and decapitated. The brain was removed rapidly and kept in ice-cold sucrose-containing artificial cerebrospinal fluid (sucrose-aCSF) oxygenated with 95% O_2_-5% CO_2_, containing (in mM) 200 sucrose, 3 KCl, 2 CaCl_2_, 2 MgCl_2_, 26 NaHCO_3_, 1.25 NaH_2_PO_4_, and 10 D-glucose, at pH ∼7.40. The brainstem was isolated from the rest of the brain and trimmed to a pontine tissue block. Transverse pontine sections (300 μm) containing the LC were obtained with a vibratome (Series 1000, The Vibratome Company, St. Louis, MO) in sucrose-aCSF. The slices were collected in oxygenated normal aCSF with 124 mM NaCl substituted for sucrose. The slices were then recovered at 33°C for 1 h and kept at room temperature until recording. Individual slice used for recording was transferred to a recording chamber that was perfused with oxygenated aCSF at a rate of 1 ml/min and maintained at 31–35°C.

### Immunohistochemical Analysis

For immunohistochemistry study, the mice were anesthetized and perfused with 4% (w/ v) paraformaldehyde. The brain was then removed, kept in the fixative overnight, and then cut transversely into 100 μm sections with a cryostat (Leica, Wetzlar, Germany).

Sections containing the LC were prepared for dopamine-β-hydroxylase (DBH)-immunostaining and detected with a fluorescently labeled streptavidin. Briefly, the floating sections were incubated with primary antibody against biotin DBH (Immunostar, 1:2000). The sections were then exposed to Alexa Fluor® 594 conjugate with streptavidin (Molecular Probes, Life Technologies, USA). Immunoreactive cells were visualized using fluorescence microscopy with excitation at 594 nm and emission at 610 nm (red) (Zeiss Axio Examiner, Jena, Germany).

### Optostimulation of GABAergic neurons

GABAergic neurons were detected in brain slices with YFP expression using fluorescence microscopy in excitation at 510 nm and emission at 520 nm (green). Optostimulation was performed by using a xenon light source with high-speed switcher (Lambda GD-4, Sutter Instruments, Novato, CA). The light source was connected to the incident-light illuminator port of the microscope, and delivered blue light through a 470 nm bandpass filter. The 10 ms pulse trains were triggered by the Digitimer D4030 pulse generator (Digitimer Ltd, UK). The latency of light-evoked action potentials was measured from the onset of the light to the onset of action potentials.

### Electrophysiology

The whole-cell recordings were performed in brain slices with cell visualization using a 40X water-immersion lens in the Zeiss Axioskop 2 microscope and a near-infrared charge-coupled device (CCD) camera. Patch pipettes were pulled with a pipette puller (Model P-97, Sutter Instruments). The pipette resistance was 3–5 MΩ. The internal (pipette) solution for current-clamp recording contained (in mM) 130 K gluconate, 10 KCl, 10 HEPES, 2 Mg-ATP, 0. 3 Na-GTP and 0. 4 EGTA (pH 7. 3). The internal pipette solution for voltage-clamp contained (in mM) 135 CsCl, 2 MgCl_2_, 2 Mg-ATP, 1 Na-GTP, 10 HEPES (pH 7. 30). The aCSF solution was applied to the bath, containing (in mM) 124 NaCl, 3 KCl, 1. 3 NaH_2_PO_4_, 2 MgCl_2_, 10 D-glucose, 26 NaHCO_3_, 2 CaCl_2_ (pH 7. 4 with 95% O_2_ and 5% CO_2_). The slices were perfused with the external solution continuously with superfusion of 95% O_2_ and 5% CO_2_ at 33°C. The temperature was maintained by a dual automatic temperature control (Warner Instruments). In current-clamp, only neurons with stable resting membrane potentials (V_m_) more negative than -40 mV and action potential amplitude over 65 mV were used in the studies. These cells were usually recorded for >45 min, a time period that was adequate for our experimental protocol.

In voltage-clamp recording, the light-evoked and spontaneous GABA_A_ receptor-mediated inhibitory post-synaptic currents (IPSCs) were pharmacologically isolated by addition of the α-amino-3-hydroxy-5-methyl-4-isoxazolepropionic acid (AMPA) receptor antagonist 6-cyano-7-nitroquinoxaline-2, 3-dione (CNQX, 10 μM), the *N*-methyl-D-aspartate (NMDA) receptor antagonist DL-2-Amino-5-phosphonopentanoic acid (DL-APV, 10 μM), and the glycine receptor antagonist strychnine (1 μM) to the external solution. Recorded signals were amplified with an Axopatch 200B amplifier (Molecular Devices, Union City, CA), digitized at 10 kHz, filtered at 2 kHz using the low-pass filter, and collected with the Clampex 8.2 data acquisition software (Molecular Devices).

The electrophysiological data were analyzed with Clampfit 10. 3 software (Molecular Devices) and the Mini Analysis Program 6.0.7 software (Synaptosoft Inc. New Jersey, USA). Data are presented as means ± SE. Statistical analysis of other parameters was performed using the ANOVA, the two-tailed Student’s *t*-test or the Mann-Whitney test. Difference was considered significant when *P* ≤ 0.05.

## Results

### GABAergic interneurons in the LC

**Fig 1:**
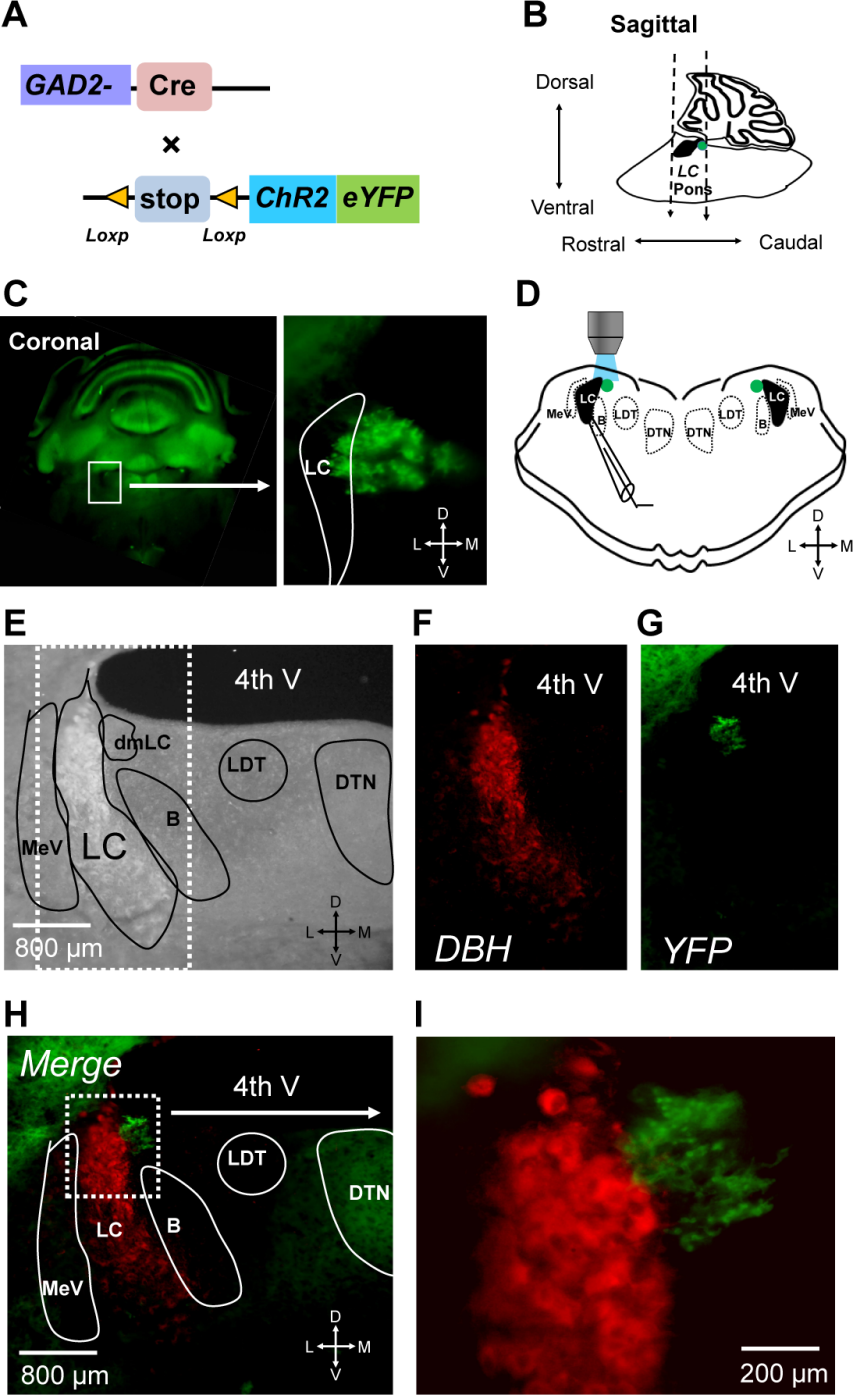
(A) Transgenic strategy for expression of ChR2 (H134R) in GABAergic neurons. (B) A sagittal schematic representation of the brain region used in this study (left). Black area: LC region; Green contours: GABAergic interneurons. (C) The coronal section of a mouse brain slice containing LC region (left). High magnification image showing that a group of YFP fluorescence-positive GABAergic neurons is located immediately to LC. (D) Left schematics showing a brain slice with patch clamp electrode on LC neuron. The dmLC area labeled with green color for light excitation. (E) Representative photomicrographs of transverse pontine sections containing the LC neurons from a brain slice of transgenic mice. Transverse pontine section presented was obtained from transgenic mice (E). The framed areas showed the typical large LC neurons, distributed along the lateral floor of 4^th^ ventricle. The same section was incubated with Anti-DBH (conjugated with biotin) antibody, followed by Alexa Fluor® 594 (conjugated with streptavidin) (F). YFP-positive GABAergic neurons were identified by green fluorescence by illumination at 470 nm (G). (H) Merged image of GABAergic neurons (E, green) and LC neurons (F, red). (I) Enlargement of the framed area in C, showed closely intermingled GABAergic neurons and LC neurons. LC, locus coeruleus; B, Barrington’s nucleus; dmLC, dorsomedial locus coeruleus; DTN, dorsal tegmental nucleus; LDT, dorsolateral tegmental nucleus, MeV, mesencephalic trigeminal tract nucleus. The location of each region was manually annotated using the Allen brain atlas representations of the mouse coronal section containing LC region (Bregma: -5.655mm) [17].

To determine the distribution of GABAergic interneurons in the LC region, we expressed channelrhodopsin (ChR2) in a tandem with yellow fluorescent protein (YFP) in GABAergic neurons driven by glutamic acid decarboxylase 2 (GAD2) promoter (Fig 1A). With respect to the LC labeled by DBH-immunostaining in transverse slices containing LC neurons, an isolated group of YFP-positive neurons was identified (Fig 1B). These YFP-positive neurons were clustered as a small and roughly sphere-shape region of ~300μm in diameter seen in only one 300μm brain slice or split in two adjacent slices. This group of cells was located immediately below the 4^th^ ventricle dorsomedial to the LC and just dorsal to Barrington’s nucleus (Fig 1C and 1E) [12]. Some of the YFP-positive cells overlapped with DBH-positive neurons in the LC, although no cells with double labeling of both DBH (Fig 1F) and YFP were found (Fig 1G). In higher magnification, these YFP-positive cells had rather small soma in comparison to LC neurons (Fig 1I). According to the location relative to the LC, we named this group of GABAergic neurons the dorsomedial LC (dmLC) nucleus.

### Electrophysiology properties of the dmLC GABAergic interneurons

**Fig 2:**
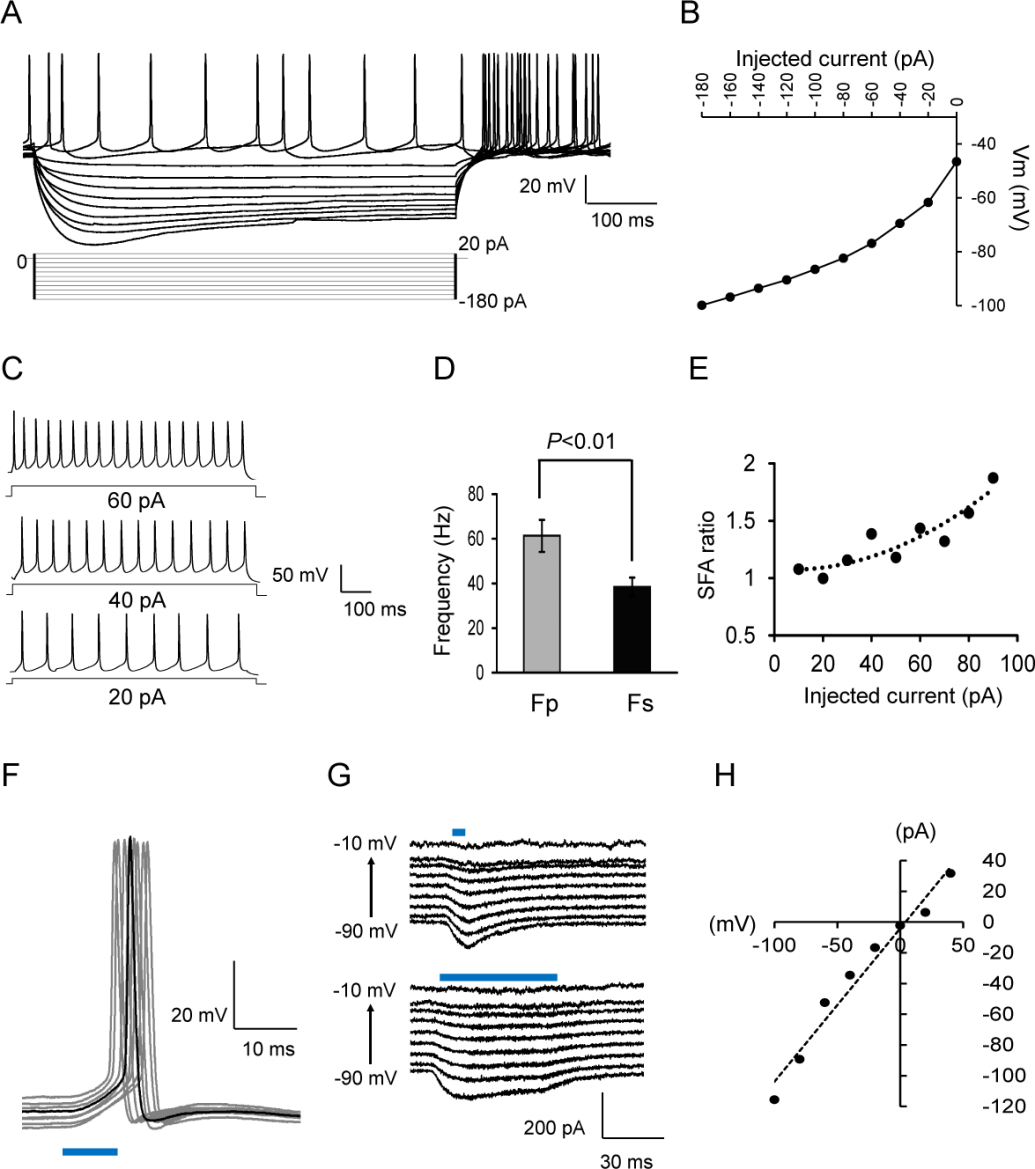
The electrophysiology properties of the GABAergic neurons in dmLC area. (A) Whole-cell clamp recording of the GABAergic neurons in dmLC area. The membrane potential was held at 0 mV and stepped from -180 to 20 mV every 1s. (B) I-V curve of GABAergic neurons in dmLC area. (C) Spike frequency adaptation (SFA) properties of GABAergic neurons in dmLC area, accompanied with depolarization. (D) The firing frequency in peak state (Fp) and in the steady state (Fs) was compared and represented in bar graph. Data are presented as means ± SE (n = 18 cells, six animals). (E) The ratio of Fp/Fs were calculated following the current injection steps (from 10–90 pA). (F) The optical responses to 10 ms 470 nm optostimulation in both current-clamp recording. Black trace, a typical individual trace. (G) Voltage-clamp recording from GABAergic neuron in dmLC, where strong inward photocurrent are evoked by 5 (top) and 50 ms (bottom) light pulses. (H) *I-V* relationship of light-evoked current in GABAergic neuron.

Since the YFP-positive neurons in the dmLC area could be identified using epifluorescence microscopy, electrophysiological properties of these neurons were studied in whole-cell patch clamp recordings. These dmLC neurons had averaged membrane potentials -49.2 ± 2.2 mV, with input resistance 555.2 ± 34.1 MΩ, and capacitance 19.0 ± 0.8 pF (n = 18 cells, from six animals). They displayed a large sag potential (23.9 ± 1.9 mV, n = 18 cells) with post-inhibitory rebound (PIR) immediately after hyperpolarization (Fig 2A). All these neurons showed strong inward rectification with hyperpolarizing currents injections (Fig 2B).

Most (14 out of 18 neurons) of the dmLC neurons fired spontaneously with fast firing frequency (10.9 ± 1.1 Hz, n = 14) in comparison to LC neurons (4.3 ± 0.5 Hz, n = 15, P<0.01). With a series of depolarizing current injections, the firing frequency of these neurons increased with each depolarization (Fig 2C). In response to the same depolarizing pulse, the firing frequency of the first two action potentials (Fp) was significantly higher than those in the steady state (Fs), which were 61.3 ± 7.1 Hz and 38.4 ± 4.2 Hz, n = 16 (P < 0.01) (Fig 2D), respectively. The spike-frequency adaptation (SFA) indicated as Fp/Fs ratio increased with more depolarizing currents (Fig 2E).

Exposure of the dmLC neurons to10 ms blue light (470 nm) evoked rapid depolarization followed by action potential in current clamp (Fig 2F). In voltage clamp, the light pulse produced strong inward currents that showed modest adaptation over a long period of light exposure (Fig 2G). The inward light-evoked current had a reversal potential around 0 mV when the I-V relationship was tested with a series of command steps (Fig 2H).

### Evidence for the inhibition of LC neurons by dmLC GABAergic interneurons

**Fig 3:**
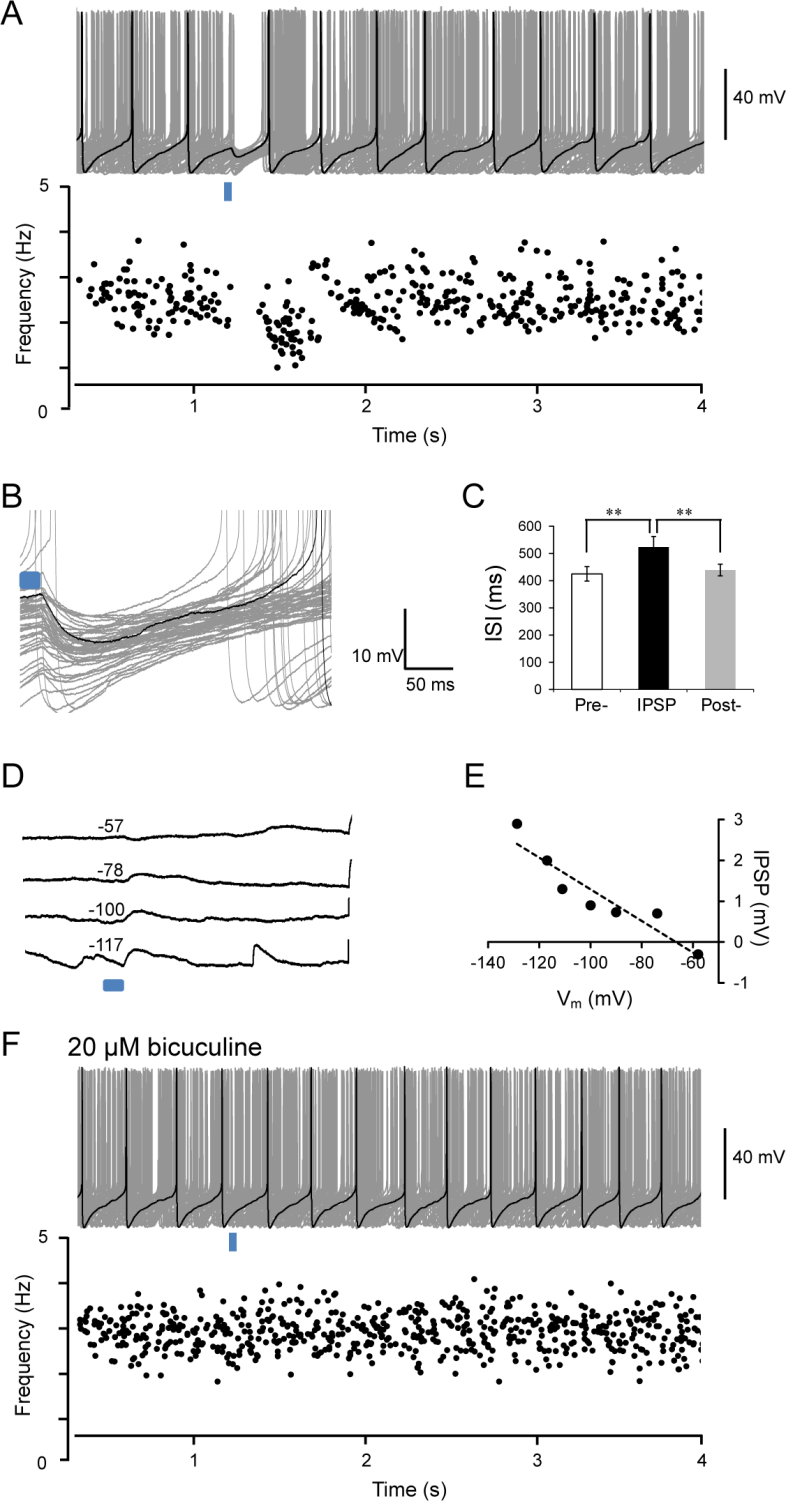
Response of LC neurons to the inhibitory postsynaptic potentials (IPSPs) that were evoked by optostimulation of these dmLC GABAergic neurons. (A) LC neuronal spontaneous firing activity was reset by light-evoked IPSP (top). The effect of light-evoked IPSPs on LC neuron firing activity was measured in instantaneous firing frequency histogram (bottom) Light pulse: 10 ms. (B) Light-evoked IPSPs are better seen at extended scale of A. (C) The effect of light-evoked IPSPs on inter-spike interval (ISI) were represented by bar graph. (D) The light-evoked IPSP is strongly influenced by membrane potential of LC neuron. (E) The equilibrium potential for IPSP close to -60 mV. (F) A GABA_A_ receptor antagonist, 20 μM bicuculline completely abolished light-evoked IPSPs of LC neuron. Data are presented as means ± SE (n = 15 cells, three animals).

To gain some perspectives about the interaction of these dmLC cells with LC neurons, we studied the response of LC neurons to optostimulation. The LC neurons were densely packed along the lateral floor of 4^th^ ventricle at dorsolateral pons (Fig 1D and 1F). In current clamp, all LC neurons studied exhibited typical electrophysiology properties as we have seen in our previous studies [7, 8], including a less negative V_m_, inward rectification, spontaneous firing, no PIR and strong delayed excitation (Data not shown). LC neurons are endogenous pacemakers that display tonic spontaneous firing activity resulting from their intrinsic membrane properties [13] Focal stimulation of the dmLC by 470 nm blue light in a small area (~300 μm in diameter) strongly inhibited firing activity of LC neurons (Fig 3A). Inhibitory postsynaptic potentials (IPSPs) were clearly seen in the LC neurons (V_m_ -45.1 ± 1.5 mV, n = 12 cells, three animals) following each light pulse (10 ms) stimulation (Fig 3B). With step current injections, the IPSPs elicited by optostimulation of dmLC neurons changed their amplitudes gradually. In the current-voltage (I-V) plot, the IPSPs showed linear conductance with reversal potential around -60 mV consistent with the Cl^-^ equilibrium potential in our recording solutions (Methods, Fig 3D and 3E).

Without current injection, optostimulation produced hyperpolarization that efficiently inhibited LC firing activity (Fig 3A). With these light-evoked IPSPs, the firing frequency of LC neurons was inhibited instantaneously (Fig 3A) as indicated by an expansion of the inter-spike interval; action potentials were aborted when concurrent with the light pulse; and the LC neuronal pacemaking activity was reset (Fig 3A and 3C). Application of the selective GABA_A_ receptor antagonist bicuculline (20 μM) completely abolished light-evoked IPSPs (Fig 3F). These results indicate that these dmLC neurons are indeed GABAergic neurons.

**Fig 4:**
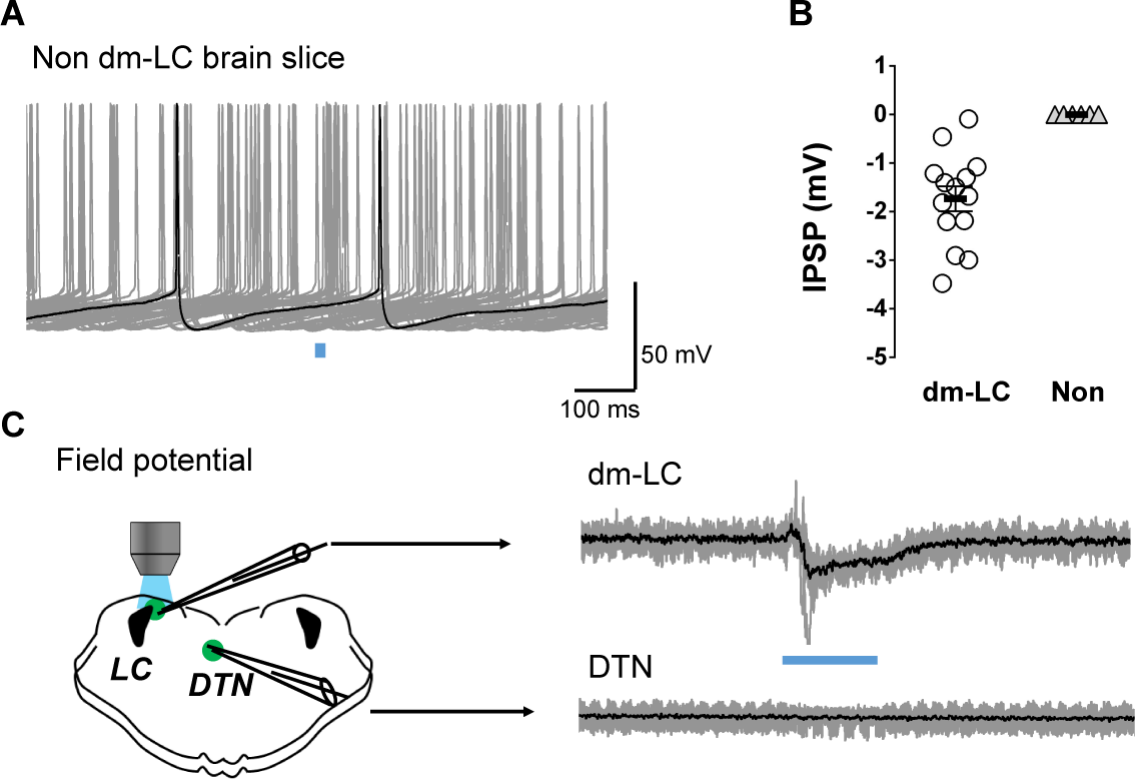
(A) In the brain slices without dmLC GABAergic neurons, 10 ms blue light pulse failed to elicit IPSP in LC neurons. (B) Quantification of GABAergic IPSPs in LC neurons of dmLC (circle, n = 14 cells of 6 slices) and non dmLC brain (triangle, n = 9 cells of 5 slices) slices. (C) Blue light (50 ms) evoked field potentials when the extracellular recording electrode was positioned in the dmLC but not in the DTN.

Such IPSPs were not seen in brain slices where the dmLC was missing (Fig 4A and 4B). Beside the dmLC, another group of GAD2-postive neurons was found in the dorsal terminal accessory optic nuclei (DTN) region within the brain slices. Application of blue light to dmLC area failed to evoke any antidromic field potential in the DTN neurons (Fig 4C).

**Fig 5:**
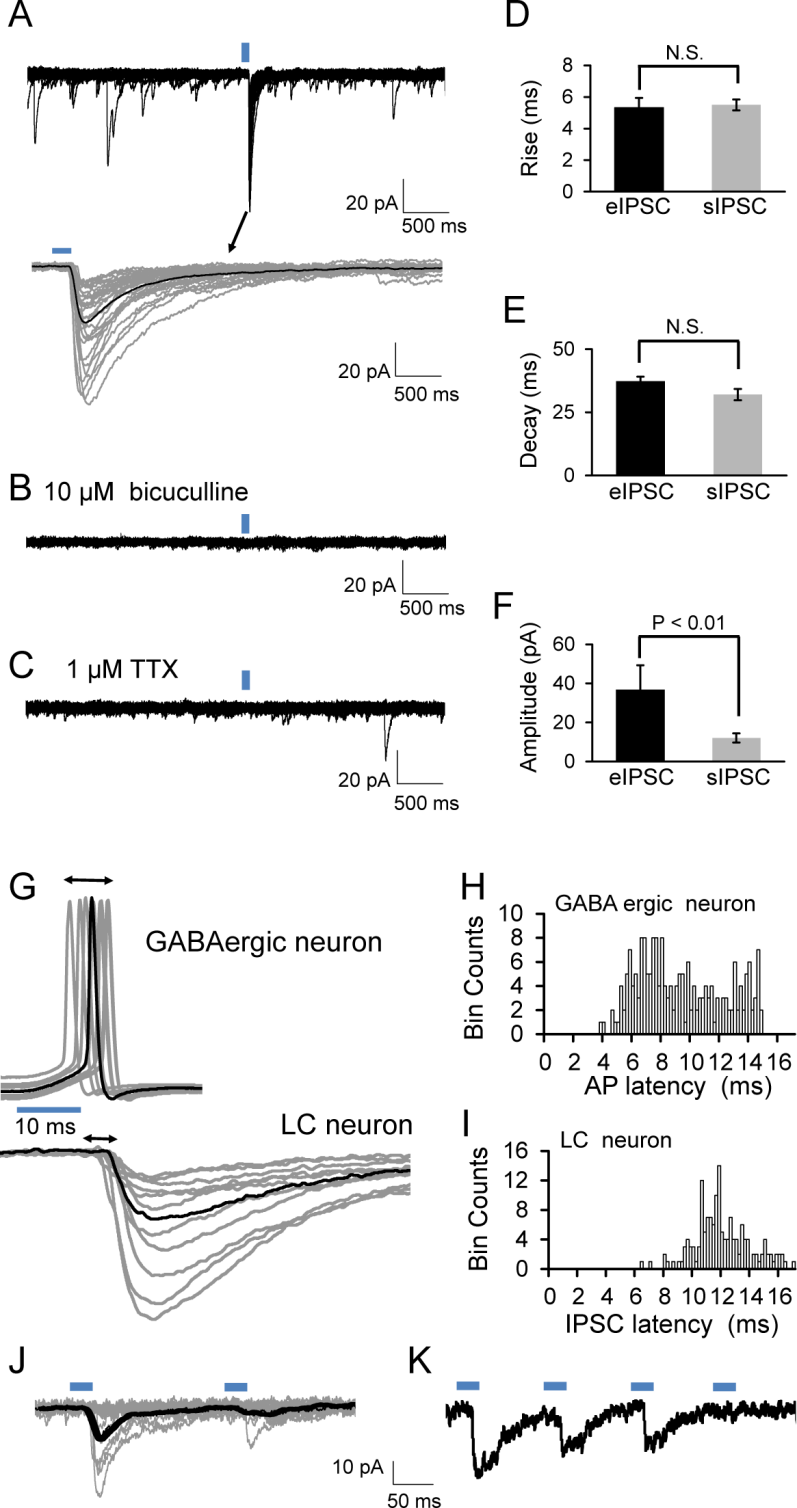
Postsynaptic responses to GABAergic neurons in LC region. Same Cl^*‒*^ concentrations were applied to the internal peptide and bath solutions for whole cell voltage clamp. The GABA_A_-mediated inward currents were recorded at a holding potential of –70 mV. All glutamate and the glycine receptors were pharmacologically blocked by application of 10 μM CNQX, 10 μM APV, and 1 μM STY to bath solution. (A) The IPSCs in LC neurons produced by applying 10 ms 470 nm light to the dmLC GABAergic neurons (upper). Lower panel displays enlarged IPSCs from upper trace. The averaged trace (black) was carried out within 50 individual traces (gray). Perfusion with 20 μM bicuculine (B) or 1 μM tetrodotoxin (TTX) (C) eliminated the IPSCs. The rise time (D), the decay time (E) and the amplitudes (F) of light evoked (eIPSCs) and spontaneous IPSCs (sIPSCs) were compared in bar graph. (G) Representative recording of light-evoked action potentials in a presynaptic GABAergic neuron aligned to light-evoked IPSCs in a LC neuron (black trace, average of 30 individual traces). Histograms of action potentials (H) and IPSCs (I) latencies. (J) Twenty consecutive traces of IPSCs elicited by pair light-stimulation of GABAergic neurons. (K) The multiple light-stimulation evoked IPSCs in a LC neuron.

In voltage-clamp with symmetric Cl^*‒*^ concentrations applied to the pipette and bath solutions, GABA_A_-receptor mediated inward currents were recorded in LC neurons at a holding potential of –70 mV when all glutamate and glycine ionotropic receptors were pharmacologically blocked by application of CNQX (10 μM), APV(10 μM) and strychnine (1 μM). Under this condition, applying 10 ms light pulses to the dmLC elicited large inward currents with symmetric concentrations of Cl⁻ in LC neurons (Fig 5A). The rise time and decay time of these evoked IPSCs are similar to spontaneous IPSCs (Fig 5D and 5E). The amplitude of light-evoked IPSCs were much larger and highly variable (from 7.5 to 190 pA), likely due to spontaneous firing activity and the refractory period of the GABAergic neurons (Fig 5F). The GABA_A_ receptor antagonist bicuculline (10 μM) completely abolished the light-evoked IPSCs in the LC neuron (Fig 5B). In addition, the light-evoked IPSCs were completely blocked by the application of voltage-gated Na^+^ channel antagonist tetrodotoxin (TTX, 1 μM), suggesting these light-evoked IPSCs are action potential dependent (Fig 5C).

Onset latencies of the light-evoked action potentials in GABAergic neurons and the IPSCs of LC neurons were analyzed (Fig 5G and 5H). The latency of light-evoked action potentials in dmLC GABAergic neurons ranged from 4 ms to 15 ms (averaged 9.4 ± 0.2 ms, n = 196 traces of 7 neurons, in 3 animals) (Fig 5H), while the latency of IPSCs in LC neurons ranged from 6 ms to 17 ms (averaged 12.6 ± 0.2 ms, n = 190 traces of 7 neurons, in 3 animals) (Fig 5I). The difference between two latencies was 3–6 ms. Rise time of IPSCs (10 to 90% of the peak amplitude) were also compared between light-evoked IPSCs and spontaneous IPSC in the same cell. Of 4 cells studied, the rising time averaged 5.3 ± 0.6ms (n = 158 traces) and 5.5 ± 0.4ms (n = 92 traces) for spontaneous IPSCs and light-evoked IPSCs, respectively. Their difference was statistically insignificant. Considering the similarity of onset latency and the IPSC rising time, these IPSCs are likely to be monosynaptic.

To study the activity-dependent depression of IPSCs, pair and multiple optostimulations with 10 ms pulses were applied to the dmLC GABAergic neurons. As shown in Fig 5J, a pair of optostimulation to the GABAergic neurons caused a decrease in the amplitude of second IPSC in LC neurons. In addition, the depressed IPSCs magnitude was also observed during continuous stimulation of these presynaptic GABAergic neurons (Fig 5K), suggesting that neuronal communication characteristics including receptor desensitization and refractory period after action potentials seem to remain with optostimulation.

### Lateral inhibition among GABAergic neurons

**Fig 6:**
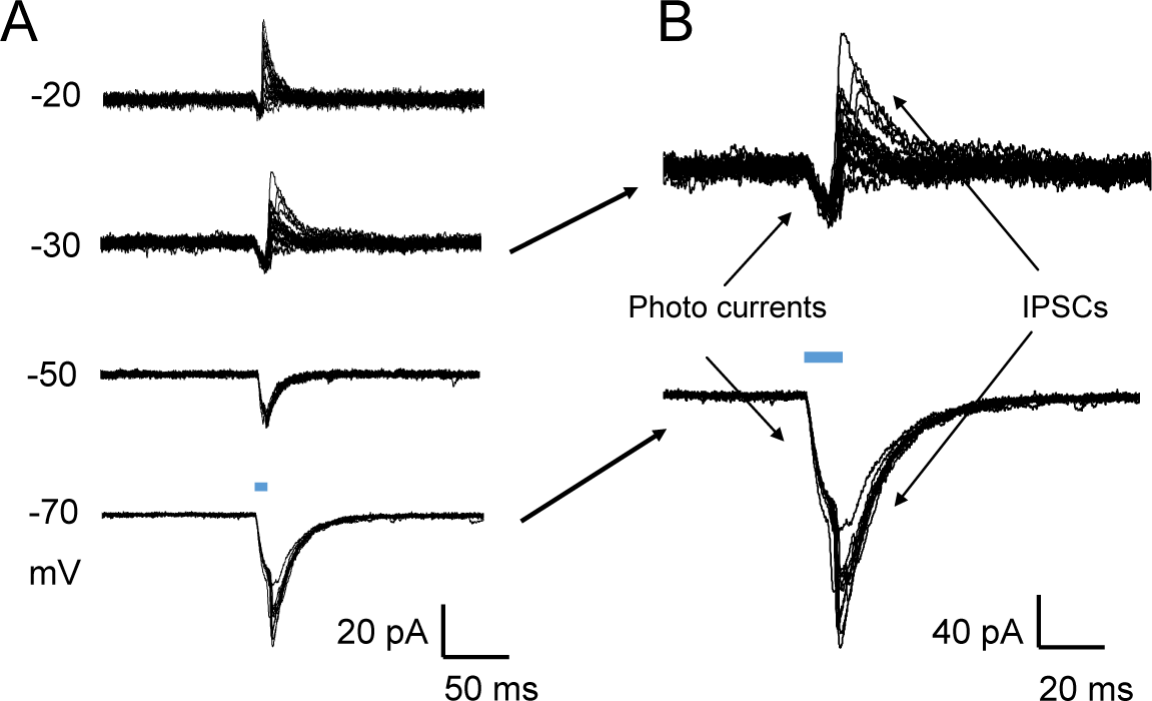
Optostimulation evoked lateral inhibition among GABAergic neurons. (A) Voltage clamp recording for light evoked inward currents. The holding potential were stepped from -20 to -70 mV. (B) Enlarged traces from holding potentials at -30 (upper) and -70 mV (lower).

In 4 out 10 GABAergic neurons, optostimulation evoked both inward and outward currents. The early inward currents had a reversal potential at 0 mV, while the reversal potential the late outward currents was close to -50 mV (Fig 6A). To distinguish the outward currents from the inward light-evoked currents, we raised the holding potentials to more depolarization so that the inward currents were reduced. Under the condition, the evoked IPSCs were clearly seen in the GABAergic neurons (Fig 6B). The onset latency (13.2 ± 0.6 ms, n = 56 traces, 4 slices) and rising time (4.5 ± 0.3 ms, n = 56 traces, 4 slices) of the IPSCs suggest that they are monosynaptic as well. In contrast, optostimulation of the dmLC did not produce any currents in the DTN neurons (Fig 4C). Thus, optostimulation of the GABAergic neurons produced monosynaptic inhibition of the same group of neurons.

## Discussion

In the present study, we took an optogenetic approach and expressed transgenically ChR2 with YFP in GABAergic neurons. In these mice, a group of GABAergic neurons was found in the dorsomedial LC region with a number of cells intermingled with LC neurons. We demonstrated the intrinsic membrane properties of these GABAergic neurons and their synaptic interaction. Also, several pieces of evidence suggest that they inhibit LC neurons monosynaptically.

Our studies in the GAD-targeted mice revealed a group of GABAergic neurons in the LC area. This small nucleus is located immediately dorsomedial to the LC and dorsal to the Barrington’s nucleus. A previous study has shown a group of loosely distributed GAD-positive neurons in the peri-LC dendritic area in rats, which was named the pericoeruleus neurons [14]. The lack of membrane electrophysiological information of peri-LC neuron does not allow us to compare these rat peri-LC neurons to mouse dmLC cells. Based on morphological results, the mouse dmLC GABAergic neurons may not be an analog of the rat peri-LC neurons for several reasons: i. Location. The mouse dmLC nucleus is located dorsomedial to the LC, while the rat pericoeruleus neurons site at ventromedial area. ii. Cell density. The dmLC nucleus is packed with YFP positive cells with clear boundaries, while the pericoeruleus cells are scattered without distinguishable nucleus boundary. iii. Interface with LC. The dmLC nucleus makes a side-by-side contact with highly packed LC neurons in the dorsal area, while the pericoeruleus cells surround the ventral corner of the LC where LC neurons are rather dispersed. In addition, the dmLC is just below the 4th ventricle with dendritic contact to the ventricle. Although several groups of GABAergic neurons are found in the pons, evidence for their intrinsic membrane properties and their contributions to local neuronal networks is still lacking. Using the optogenetic approach, the present study has revealed the intrinsic membrane properties of these GABAergic neurons. These cells have strong inward rectification, fire fast action potentials spontaneously, and show moderate SFA and PIR. With such intrinsic membrane properties as well as their anatomical location and small soma, these cells should be readily identifiable in brain slices even without special labeling.

We have found that the dmLC GABAergic neurons inhibit each other monosynaptically. Such synaptic interaction seems to be lateral inhibition. Our results favor the reciprocal inhibition between the dmLC neurons, as no disinhibition was found in LC neurons following optostimulation. The lateral inhibition of GABAergic interneurons has been shown to play an important role in maintaining neuronal rhythmic activity. Such inhibitory interactions among GABAergic neurons can reduce the total inhibitory outputs, lower the synaptic inhibition frequency, and allow PIR to take place in a short time window [15, 16].

Optostimulation of the dmLC area produces IPSPs in LC neurons, hyperpolarizing the cells and resetting their spontaneous firing activity. The IPSCs appear to be monosynaptic as their onset and decay times are the same as sIPSCs, they are not disinhibition, and their latency is too short for polysynaptic (>3 ms) neuronal involvements. Recurrent and reciprocal inhibitions of LC neurons do not seem to play a role, as we did not find any evidence for synaptic inhibition of dmLC neurons following spontaneous and evoked action potentials of LC neurons. Alternatively, these GABAergic neurons may serve as interneurons of LC to produce feed-forward inhibitions from unknown brain regions

Our results indicate that LC neuronal inhibition is mainly produced by dmLC optostimulation but not by optostimulation of the DTN and GABAergic axonal terminals for several reasons: 1) LC neuronal inhibition is not seen in brain slices where the dmLC is missing. 2) Our optostimulation is limited by the objective lens in an area of ~300 μm in diameter, and does not reach DTN 1–2 mm away. 3) Antidromic field potentials in the DTN cannot be produced by dmLC optostimulation. 4) The LC neuronal inhibition appears to be monosynaptic according to our measurements and calculation, suggesting that the effect is direct rather than disinhibition via another neuron. 5) The light-evoked IPSCs are completely blocked by the application of TTX, suggesting action potential dependence (Fig 5C). Despite these, it is still possible that light may activate axons of GABAergic neurons whose cell bodies are located in other region or no longer exist in the brain slice. Thus better and more direct evidence for monosynaptic inhibition of LC neurons by the dmLC cells is still needed, while our data showing the anatomical location and electrophysiological properties of the dmLC neurons benefits further studies.

It is known that truncated axons without soma can produce action potentials depending on the axonal length and stimulation strength. Although our results do not support such a process in LC neuronal inhibition by optostimulation as the inhibition was not seen in the absence of the dmLC in brain slices, our results cannot completely exclude this possibility. Therefore, synaptic inhibition of LC neurons by dmLC neurons is suggestive but not conclusive. More evidence is needed, especially that obtained with approaches different from optogenetics. Clearly, that is beyond the scope of this present study. Nonetheless, our demonstration of the dmLC neurons in terms of their location, somatic morphology, intrinsic membrane properties, and synaptic interactions with each other facilitates further studies of this newly identified GABAergic neurons in the LC area.

## Conclusion

A group of GABAergic neurons has been identified in the present study. These GABAergic neurons have small and distinguishable soma, form an isolated and densely packed nucleus, and are in close contact with LC neurons. Their passive and active membrane properties have been demonstrated, which are clearly different from those of LC neurons. These GABAergic neurons mutually inhibit each other by lateral inhibition. They appear to inhibit LC neurons as well as a part of local LC neuronal circuitry.
